# Loc4Lnc: Accurate prediction of long noncoding RNA subcellular localization via enhanced RNA sequence representation

**DOI:** 10.1002/qub2.100

**Published:** 2025-03-27

**Authors:** Yujia Cheng, Xiaoyong Pan, Yang Yang

**Affiliations:** ^1^ Department of Computer Science and Engineering Shanghai Jiao Tong University Shanghai China; ^2^ SJTU Paris Elite Institute of Technology (SPEIT) Shanghai Jiao Tong University Shanghai China; ^3^ Institute of Image Processing and Pattern Recognition and Key Laboratory of System Control and Information Processing Ministry of Education of China Shanghai Jiao Tong University Shanghai China

**Keywords:** lncRNA, long sequence analysis, subcellular localization

## Abstract

Long noncoding RNAs (lncRNAs) are crucial in gene regulation, chromatin architecture, and cellular differentiation, playing significant roles in various diseases and serving as potential biomarkers and therapeutic targets. Understanding their precise subcellular localization is essential for elucidating their functions in biological pathways. Current methods for predicting lncRNA subcellular localization face challenges in capturing long‐range interactions within sequences. Deep learning models often struggle with feature extraction that adequately represents these distant dependencies, leading to limited predictive accuracy. We develop Loc4Lnc, a deep learning framework for predicting lncRNA subcellular localization. The model integrates convolutional layers and transformer blocks to effectively capture both local sequence motifs and long‐range dependencies within RNA sequences, followed by classification using TextCNN. Using the RNALocate v2.0 database, we constructed a benchmark dataset covering five subcellular locations (cytoplasm, nucleus, cytosol, chromatin, and exosome). The performance of the model is evaluated against existing feature extraction methods and existing predictors. Results of the Loc4Lnc study demonstrate significant improvements in predicting lncRNA subcellular localization. The model achieved a prediction accuracy of 0.636 on an independent test set, outperforming existing methodologies. Comparative evaluations showed that it consistently surpassed traditional feature extraction methods and state‐of‐the‐art predictors, highlighting its robustness and effectiveness in accurately classifying lncRNAs across five distinct subcellular locations. Loc4Lnc effectively captures long‐range interactions and optimizes information flow between distal elements, providing an effective predictive tool for the subcellular localization of lncRNAs and laying the foundation for future research on the regulation of gene expression and cellular functions by lncRNAs.

## 1 INTRODUCTION

Long noncoding RNAs (lncRNAs) are a class of RNA molecules that are more than 200 nucleotides long and do not encode proteins; yet they play pivotal roles in a wide range of cellular functions [1]. These RNA molecules are involved in essential biological processes such as gene regulation, maintenance of chromatin architecture, and cellular differentiation [2, 3]. Unlike protein‐coding RNAs, lncRNAs interact with DNA, RNA, and proteins to influence the transcriptional and post‐transcriptional regulation of genes [4]. Their functions are diverse, ranging from modulating gene expression by acting as scaffolds for protein complexes to regulating chromatin remodeling and influencing the spatial organization of chromosomes in the nucleus [5, 6, 7].

Recent studies have highlighted the involvement of lncRNAs in a variety of human diseases including cancers, cardiovascular diseases, and neurodegenerative disorders such as Alzheimer’s disease [8, 9, 10, 11]. For instance, certain lncRNAs have been identified to contribute to tumor progression by regulating oncogenes and tumor suppressor genes [12]. Their dysregulation has also been associated with cardiovascular diseases, where they influence processes such as cardiac remodeling and atherosclerosis [13]. Given their extensive roles in disease mechanisms, lncRNAs are being investigated as potential biomarkers for early diagnosis, prognosis, and therapeutic targets [9].

The subcellular localization of lncRNAs is crucial for their specific functions. Depending on whether they are located in the nucleus, cytoplasm, or other organelles, such as mitochondria, lncRNAs can influence distinct biological pathways [14]. Nuclear lncRNAs often regulate gene transcription, chromatin structure, and epigenetic modifications, whereas cytoplasmic lncRNAs are typically involved in regulating mRNA stability, translation, and signaling pathways [4, 15]. Understanding the precise localization of lncRNAs is key to elucidating their roles in cellular mechanisms and provides insights into how they contribute to disease pathogenesis, thus holding promise for therapeutic interventions [16].

Existing computational methods for subcellular localization of lncRNA can be classified into two categories: traditional machine learning methods and deep learning methods. Traditional machine learning methods in predicting lncRNA subcellular localization often depend on extracting features based on the composition of *k*‐mer sequences [17]. This approach involves analyzing the frequency or presence of *k*‐mers (subsequences of length *k*) within the lncRNA sequences to capture sequence‐specific characteristics. Subsequently, shallow models such as support vector machines (SVM), random forests (RF), and logistic regression are employed as classifiers to predict the subcellular localization based on these features [18]. Deep learning methods, on the other hand, perform automatic feature learning that eliminates the need for manual feature extraction and allows the models to uncover complex patterns in the data. These methods have been applied successfully to lncRNA subcellular localization prediction, leveraging architectures such as convolutional neural networks, long short‐term memory networks, and more recently, transformer models [19, 20, 21].

Some computational methods for lncRNA subcellular localization have emerged in recent years. In 2018, Cao et al. presented the first subcellular location prediction algorithm in the field for sequences of lncRNAs [18]. The lncLocator uses *k*‐mer‐based features and high‐level abstraction features derived from unsupervised deep learning models, then trains RF and SVM with two different features to develop four models for a five‐class classification task. Su et al. created iloc‐lncRNA, incorporating octamer composition into general pseudo K‐tuple nucleotide composition (PseKNC), thereby improving the accuracy of predicting lncRNA subcellular locations [22, 23]. Fan et al. developed lncLocPred using sequence features, including *k*‐mer, triplet, and PseDNC, and systematic feature selection through variance thresholding, binomial distribution, and F‐score [24]. Zhou et al. presented a new computational approach for predicting the subcellular localization of ncRNAs by combining multikernel learning with a graph‐regularized k‐local hyperplane distance nearest neighbor algorithm [25].

In 2021, Lin et al. launched lncLocator 2.0, an updated predictor that uses deep learning to determine lncRNA subcellular locations from sequences for specific cell lines [26]. lncLocator‐imb, proposed in 2023, similarly focuses on the problem of classifying lncRNAs on 13 cell lines at two subcellular locations, the nucleus and the cytoplasm [21]. Another deep learning framework, DeepLncLoc, breaks down sequences into subsequences to extract pattern information which is then amalgamated for full sequence representation [19]. Bai et al. created ncRNALocate‐EL, which utilizes four distinct feature representation methods to enhance the understanding of data from multiple perspectives: syntax rules, term frequency‐inverse document frequency (TF‐IDF), TextRank, and sequence‐based features [27]. LncLocFormer proposed by Zeng et al. in 2023 uses eight transformer blocks to model long‐range dependencies in lncRNA sequences and to share information in lncRNA sequences [20].

At the same time, researchers have also attempted to transform lncRNA sequences into graphs and use graph neural networks for feature extraction. In 2023, Li et al. introduced GraphLncLoc, a novel model leveraging graph convolutional networks (GCNs) for predicting the subcellular localization of lncRNAs [28]. GraphLncLoc converts lncRNA sequences into *de Bruijn* graphs, thereby transforming the sequence classification problem into a graph classification problem. By converting lncRNA sequences into *de Bruijn* graphs, GraphLncLoc can preserve the local order information of the sequences, improving the prediction results. In 2024, Li et al. proposed SGCL‐LncLoc, an interpretable deep learning model based on supervised graph contrastive learning, building on the success of GraphLncLoc [29]. SGCL‐LncLoc introduces supervised contrastive learning, enabling the model to learn relationships between different samples by comparing the similarity within the same category and dissimilarity between different categories. It also incorporates a global attention pooling mechanism that enhances the model’s discriminative ability.

Despite the promising achievements of these existing methods, there is still potential for improvement. For feature extraction methods, transforming raw lncRNA sequences into discriminative features is the core problem, and many methods are only based on basic feature representation methods such as *k*‐mer, PseKNC, and TF‐IDF [18, 23, 27]. Although these methods are useful for identifying local sequence patterns, they often fall short of capturing the long‐range interactions that are crucial for understanding the spatial and functional dynamics of lncRNAs. In the realm of DNA/RNA sequence prediction, the consideration of these interactions is of paramount importance. Numerous elements have been experimentally validated to exert significant regulatory roles—elements such as enhancers, repressors, and insulators can influence gene expression from distances well beyond 20 kilobases (kb) [30]. This limitation results in lower predictive accuracy, especially for sequences where distant elements play a critical role in localization. Moreover, most of these methods are based on earlier versions of databases that have been iteratively updated for a long time. The classification definitions for subcellular localization across different predictors remain unclear. The inconsistency in the number of subcellular localization categories predicted by various models hinders direct comparison and evaluation of their performance, which presents an urgent challenge for constructing datasets based on new databases and for unifying classification standards.

To address these challenges, we develop a model named Loc4Lnc. This method integrates convolutional and transformer blocks to enhance feature extraction, making it more effective at capturing the complex long‐range interactions between distal elements within RNA sequences. Additionally, we utilize the latest generation dataset RNALocate v2.0, which provides a more comprehensive and up‐to‐date set of lncRNA subcellular localization records. Based on accurate subcellular localization prediction, we construct a standardized dataset comprising five distinct subcellular locations. Specifically, the model can predict cytoplasm, nucleus, cytosol, chromatin, and exosome localizations. Finally, we compare its performance on an independent test set against traditional feature extraction methods and existing methods, and our method achieves the best predictive performance.

## 2 RESULTS

### 2.1 Overview of Loc4Lnc

**FIGURE 1:**
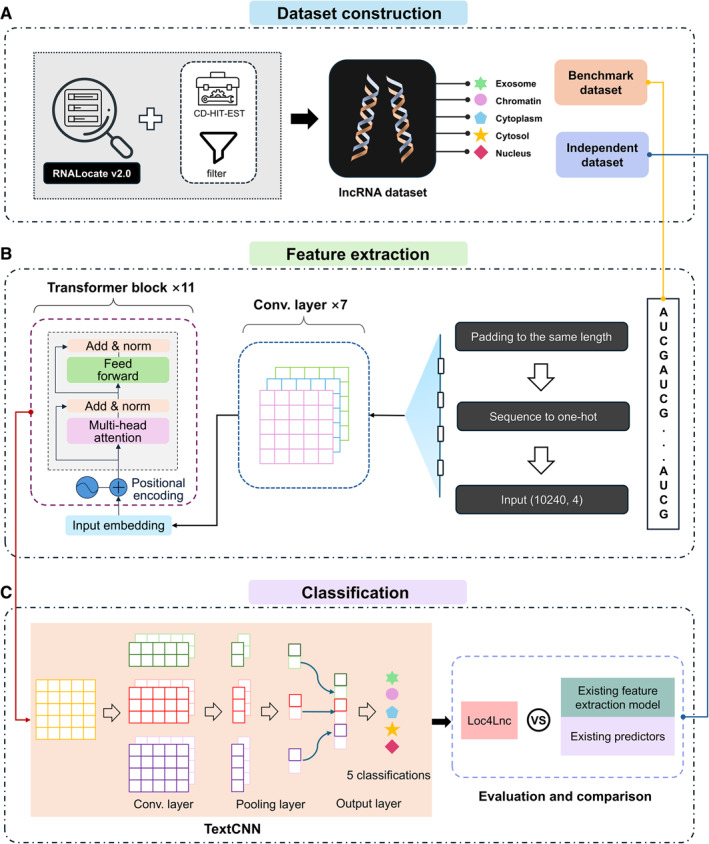
The Loc4Lnc framework with three main components: dataset construction, feature extraction, and classification. (A) Data from RNALocate v2.0 are acquired and then preprocessed to filter out five selected, predicted, and localized subcellular locations. (B) The sequences are processed through a pretrained model to obtain embedding features for different long noncoding RNAs (lncRNAs). (C) The features are fed into the TextCNN model for prediction and compared with existing feature extraction models and current predictors.

The overview of Loc4Lnc is shown in Figure 1. This process begins with the construction of a standardized lncRNA localization dataset. We utilize data from RNALocate v2.0, which performed filtering based on unique IDs, and use the CD‐HIT program for redundancy reduction [36]. As a result, we obtain a dataset of lncRNAs with five subcellular locations. The dataset is then split into training and testing sets with a ratio of 8:2.

Before being fed into the feature extraction model, the lncRNA sequence needs to be populated to a uniform length—it should be a multiple of 128—and considering the subsequent symmetric trimming, we choose an even multiple of 128. Based on the results of the screening length, we choose to fill the length to 10,240 using *N* bases to represent unknown bases. The feature extraction component utilizes a combination of 7 convolutional layers and 11 transformer blocks tailored to capture long‐range dependencies within the long sequences efficiently. The extracted feature embeddings are subsequently input into TextCNN for training on a five‐class classification task [31]. Finally, we compare the performance of the final model against other feature extraction methods combined with classifiers as well as existing predictors for lncRNA subcellular localization prediction.

### 2.2 Evaluation metrics

To evaluate the performance of our model, we use accuracy (ACC), macro precision (MaP), macro recall (MaR), and macro F1‐score (MaF1) as metrics. Accuracy is a simple and intuitive measure that is useful for balanced datasets and quick to compute. MaP highlights the exactness of the model’s predictions across all classes, making it beneficial for scenarios where false positives are costly. MaR ensures that each class is considered equally, making it essential for detecting all positive instances, especially in sensitive applications. Finally, macro F1‐score balances precision and recall, offering a comprehensive view of the model’s performance and mitigating the impact of class imbalance. These metrics collectively provide a holistic evaluation of a model’s effectiveness in multiclass classification.

(1) $$\begin{array}{c} \text{Accuracy}=\frac{\sum_{i=1}^{N}1\left(y_{i}=\hat{y_{i}}\right)}{N}, \end{array}$$

(2) $$\begin{array}{c} {\text{Precision}}_{c}=\frac{TP_{c}}{TP_{c}+FP_{c}}, \end{array}$$

(3) $$\begin{array}{c} \text{Macro}\;\text{Precision}=\frac{1}{C}\sum_{c=1}^{C}{\text{Precision}}_{c}, \end{array}$$

(4) $$\begin{array}{c} {\text{Recall}}_{c}=\frac{TP_{c}}{TP_{c}+FN_{c}}, \end{array}$$

(5) $$\begin{array}{c} \text{Macro}\;\text{Recall}=\frac{1}{C}\sum_{c=1}^{C}{\text{Recall}}_{c}, \end{array}$$

(6) $$\begin{array}{c} {F1-\text{score}}_{c}=2\cdot \frac{{\text{Precision}}_{c}\cdot {\text{Recall}}_{c}}{{\text{Precision}}_{c}+{\text{Recall}}_{c}}, \end{array}$$

(7) $$\begin{array}{c} \text{Macro}\;F1-\text{score}=\frac{1}{C}\sum_{c=1}^{C}{F1-\text{score}}_{c}. \end{array}$$

Here, *N* is the total number of samples, *C* is the total number of classes, *y*_*i*_ is the true label of sample *i*, $\hat{y_{i}}$ is the predicted label of sample *i*, and 1(⋅) is the indicator function. *TP*_*c*_ is the number of true positives for class *c*, *FP*_*c*_ is the number of false positives for class *c*, and *FP*_*c*_ is the number of false negatives for class *c*.

### 2.3 Comparison with existing feature extraction models

In this section, we compare Loc4Lnc with existing feature extraction methods combined with traditional classifiers, including the following feature extraction methods:

1. ***K*‐mer composition**: A method that represents sequences by breaking them down into *k*‐sized subsequences, allowing for the capture of local sequence composition.
2. **Word2vec [** 32 **]**: Encoding lncRNA sequences with word2vec involves firstly breaking down the RNA sequences into *k*‐mers subsequences, treating these as “words”. These *k*‐mers are then fed into the word2vec model to generate vector representations, capturing the contextual relationships between different sequence parts. This process transforms biological sequences into a numerical format, facilitating their analysis through computational methods.
3. **DNABERT [** 33 **]**: DNABERT adapts the bidirectional encoder representations from transformers (BERT) model for DNA sequence analysis. It overcomes traditional challenges by leveraging both upstream and downstream contexts, enabling accurate predictions of regulatory elements. DNABERT’s pretraining on the human genome allows for fine‐tuning on specific tasks, achieving state‐of‐the‐art performance in identifying promoters, splice sites, and transcription factor‐binding sites.

We assess these methods working with different classifiers such as multilayer perceptron (MLP) and SVM. We use the pretrained DNABERT model available on GitHub (jerryji1993/DNABERT). For the classification task, the previously constructed benchmark dataset is split into training and test sets in the ratio of 8:2. All models are trained on the training set and tested using the full independent test set. For word2vec and DNABERT‐based methods, we experiment with different *k*‐mer settings. Choosing an odd *k*‐mer length ensures a unique central nucleotide, which helps maintain directionality and improves sequence orientation during pattern recognition and alignment, so we use 3‐mer and 5‐mer versions. This distinct center reduces ambiguity that can arise from reverse complement overlaps in even *k*‐mers, enhancing algorithm performance and analytical accuracy.

The results are presented in Table 1. Loc4Lnc outperforms all the baseline methods, achieving an accuracy of 0.636. However, when examining other methods like word2vec, several notable limitations emerge. Word2vec struggles to effectively capture long‐range dependencies within RNA sequences because of its sliding window approach primarily focusing on the local context. This limitation is particularly problematic given that key features in RNA sequences often rely on long‐distance base pair interactions. Furthermore, compared to text data, the complex and diverse base composition and patterns in RNA sequences limit word2vec's generalization ability, potentially leading to insufficient representation of sequence features. Additionally, the embedding vectors generated by word2vec are largely based on statistical information, lacking integration with specific biological contexts that can result in the deficit of intuitive meaning and interpretability in biological analyses.

**TABLE 1:** Performance of Loc4Lnc and the existing feature extraction models.

Model	MaP	MaR	MaF1	ACC
5‐Mer + MLP	0.192	0.204	0.459	0.459
word2vec (3‐mer) + MLP	0.208	0.236	0.357	0.357
word2vec (5‐mer) + MLP	0.259	0.263	0.259	0.538
DNABERT (3‐mer) + MLP	0.530	0.559	0.477	0.483
DNABERT (3‐mer) + SVM	0.398	0.532	0.372	0.409
DNABERT (5‐mer) + MLP	0.504	0.588	0.475	0.547
DNABERT (5‐mer) + SVM	0.427	0.617	0.459	0.546
**Loc4Lnc**	**0.569**	**0.647**	**0.581**	**0.636**

*Note*: Bold values denote the best results.

Abbreviations: ACC, accuracy; MLP, multi‐layer perceptron; SVM, support vector machines.

Similarly, although DNABERT presents a more advanced approach, it requires substantial computational resources, especially when dealing with long RNA sequences, which can pose significant challenges in large‐scale analyses or when processing sequences typical of lncRNAs, often ranging from several hundred to several thousand nucleotides in length. At the same time, according to the method description provided by DNABERT, it can basically process sequences with a length of 512, so when long sequences are encountered, it is necessary to ensure that the sequence length is a multiple of 512, leading to potential inefficiencies or the need for sequence truncation, which could further impact the quality of the extracted embeddings.

### 2.4 Comparison with existing predictors

In addition to the investigation on the efficacy of feature representation for lncRNA subcellular localization, we also compare the performance of Loc4Lnc with existing state‐of‐the‐art predictors, including lncLocator (csbio.sjtu.edu.cn/bioinf/lncLocator), DeepLncLoc (bioinformatics.csu.edu.cn/DeepLncLoc), GraphLncLoc (csuligroup.com:8000/GraphLncLoc), and LncLocFormer (csuligroup.com:9000/LncLocFormer), and utilize their web servers to conduct the prediction.

1. **LncLocator** uses an ensemble learning approach, combining random forest and SVM models trained on *k*‐mer and autoencoder‐extracted features. It integrates outputs using a stacked neural network and applies a supervised over‐sampling method to address the class imbalance, achieving improved predictive performance [18].
2. **DeepLncLoc** uses a subsequence embedding method to retain sequence order. It divides sequences into subsequences, encodes them using a word2vec‐like approach, and employs TextCNN to extract high‐level features, and an average pooling layer further compacts the feature representation for final prediction [19].
3. **GraphLncLoc** transforms lncRNA sequences into *de Bruijn* graphs to capture local order information and sequence patterns. The nodes in the graph represent 4‐mer units and their contextual relationships are used as edges. Node features are generated using word2vec embeddings. The GCN is then applied to learn high‐level graph features that are fed into a fully connected layer for subcellular localization prediction [28].
4. **LncLocFormer** is a transformer‐based model that uses subsequence embeddings via word2vec, followed by eight transformer blocks to model long‐range dependencies. A localization‐specific attention mechanism is applied to capture distinct patterns for different subcellular localizations, enhancing prediction for multiple compartments simultaneously [20].

In addition to differences in feature extraction methods and model construction, these predictors vary in their label settings. This variation is reflected in three aspects: the number of classification categories, the selection of specific subcellular locations, and the source database of the dataset. Table 2 shows the supported location labels for each predictor. We conducted a targeted and fair comparison of these predictors based on their different classification settings. LncLocator and DeepLncLoc can both predict five subcellular locations: cytoplasm, nucleus, cytosol, ribosome, and exosome. GraphLncLoc predicts four locations: cytoplasm, nucleus, ribosome, and exosome. LncLocFormer covers four subcellular locations: cytoplasm, nucleus, chromatin, and insoluble cytoplasm. Loc4Lnc can predict five locations: cytoplasm, nucleus, cytosol, exosome, and chromatin. In terms of the selection of source databases, DeepLncLoc, lncLocator, and GraphLncLoc all utilize RNALocate v1.0, whereas LncLocFormer and Loc4Lnc use RNALocate v2.0.

**TABLE 2:** Comparison of predictable subcellular locations of Loc4Lnc and existing predictors.

Model	Subcellular locations	# of classes
lncLocator	Cytoplasm, nucleus, cytosol, ribosome, and exosome	5
DeepLncLoc	Cytoplasm, nucleus, cytosol, ribosome, and exosome	5
GraphLncLoc	Cytoplasm, nucleus, ribosome, and exosome	4
LncLocFormer	Cytoplasm, nucleus, chromatin, and insoluble cytoplasm	4
Loc4Lnc	Cytoplasm, nucleus, cytosol, exosome, and chromatin	5

Therefore, for a comprehensive comparison, we design three tasks according to the common labels shared by each of the four predictors and Loc4Lnc.

1. **Task 1**: Predicting the four subcellular locations of the cytoplasm, nucleus, cytosol, and exosome.
2. **Task 2**: Predicting the three subcellular locations of cytoplasm, nucleus, and exosome.
3. **Task 3**: Predicting the three subcellular locations of cytoplasm, nucleus, and chromatin.

For testing and performance comparison in the corresponding tasks, we randomly sample 20 records from each of the five subcellular locations in the independent test sets we constructed, resulting in 80, 60, and 60 test samples for the three tasks, respectively. The performance results for each task are shown in Table 3.

**TABLE 3:** Performance of Loc4Lnc and existing predictors on Tasks 1, 2, and 3.

Task	Model	MaP	MaR	MaF1	ACC
1	lncLocator	0.325	0.260	0.252	0.257
	DeepLncLoc	0.204	0.255	0.226	0.253
	**Loc4Lnc**	**0.728**	**0.662**	**0.674**	**0.662**
2	GraphLncLoc	0.590	0.406	0.386	0.414
	**Loc4Lnc**	**0.683**	**0.650**	**0.652**	**0.650**
3	LncLocFormer	0.534	0.517	0.518	0.517
	**Loc4Lnc**	**0.857**	**0.583**	**0.648**	**0.583**

*Note*: Bold values denote the best results.

Abbreviation: ACC, accuracy.

From Table 3, it is evident that Loc4Lnc (ACC = 0.662) significantly outperforms both lncLocator (ACC = 0.257) and DeepLncLoc (ACC = 0.253) on Task 1. This marked improvement highlights the effectiveness of Loc4Lnc in accurately predicting RNA subcellular localization. A closer analysis of the data reveals that the distributions of individual subcellular locations differ considerably between the RNALocate v1.0 and v2.0 databases. Because lncLocator and DeepLncLoc were trained on the earlier version, RNALocate v1.0, their generalization ability on the updated v2.0 database is notably limited, leading to a significant drop in accuracy when applied to the latter.

In addition to its superior performance on Task 1, Loc4Lnc also demonstrates clear advantages in Tasks 2 and 3. Specifically, Loc4Lnc achieves an accuracy of 0.650 in Task 2 and 0.583 in Task 3, surpassing the performance of GraphLncLoc (ACC = 0.414) and LncLocFormer (ACC = 0.517). GraphLncLoc uses a fixed *k* value to construct the *de Bruijn* graph, which restricts its ability to capture long‐range dependencies in lncRNA sequences. This limits the model’s flexibility in representing various sequence patterns and motifs. Although Loc4Lnc and LncLocFormer perform similarly in terms of accuracy and MaR, Loc4Lnc exhibits a substantial advantage in MaP and MaF1, metrics that are crucial for evaluating the model’s precision and balance in predicting across different classes. Although both Loc4Lnc and LncLocFormer are transformer‐based models, Loc4Lnc uses a model that is pretrained on a comprehensive dataset of transcriptional and epigenetic profiles (e.g., chromatin states and transcription factor binding) which provides a richer context for downstream tasks like lncRNA localization. Loc4Lnc uses custom relative positional encodings which help in better distinguishing between nearby and distant regulatory elements. This positional encoding strategy allows for precise modeling of the spatial relationships between different sequence regions, enhancing the prediction accuracy compared to standard positional encodings in LncLocFormer.

Upon further examination of LncLocFormer’s performance in Task 3, it becomes apparent that a significant portion of its predictions are skewed toward nucleus or chromatin locations. These categories are heavily represented in the training set, suggesting a potential model bias toward the majority of classes. This bias is problematic because it results in a performance disparity, particularly in MaP and MaF1, as these metrics are designed to assess a model’s precision and its ability to accurately identify true positives across both majority and minority classes. LncLocFormer’s tendency to overfit to the more common classes without adequately capturing the features of rarer classes leads to reduced sensitivity and lower performance on smaller less represented categories. In contrast, Loc4Lnc’s ability to recognize and integrate more information about long‐range interactions within RNA sequences enables it to achieve higher accuracy and more balanced performance across different subcellular locations. This capability not only underscores Loc4Lnc’s robustness as a predictive model but also confirms its superiority over previous predictors, particularly in its capacity to generalize effectively across diverse and complex datasets.

## 3 DISCUSSION

Loc4Lnc is a novel deep learning framework designed to accurately predict the subcellular localization of lncRNAs. Loc4Lnc’s architecture integrates seven convolutional layers and 11 transformer layers, enabling multi‐scale feature extraction. Convolutional layers capture local motifs and patterns, whereas transformer layers model long‐range dependencies, allowing the model to comprehensively represent the sequence features needed for accurate prediction and make it robust for distinguishing complex RNA patterns. The use of custom relative positional encodings allows Loc4Lnc to distinguish between nearby and distant elements within RNA sequences more accurately.

Loc4Lnc is trained on the most up‐to‐date RNALocate v2.0 database, which includes more accurate and diverse lncRNA subcellular localization data compared to earlier datasets. We construct a benchmark dataset containing five subcellular locations (cytoplasm, nucleus, cytosol, chromatin, and exosome) based on the v2.0 database. This ensures that the model benefits from recent advancements in RNA research, leading to improved generalization across various lncRNA types and subcellular compartments.

Compared to traditional models and state‐of‐the‐art predictors, such as LncLocator, DeepLncLoc, GraphLncLoc, and LncLocFormer, Loc4Lnc consistently achieves superior predictive performance across multiple evaluation metrics. Loc4Lnc supports prediction across five distinct subcellular locations (cytoplasm, nucleus, cytosol, chromatin, and exosome), showcasing better classification performance than other models that typically predict fewer locations. This multiclass prediction capability makes it versatile for comprehensive subcellular localization analysis.

Despite its strengths, Loc4Lnc has some limitations. Although the adoption of the pretrained model Enformer can partially alleviate the issue of computational complexity, the complexity still grows exponentially with the sequence length. Additionally, the predictive performance of Loc4Lnc is limited by the quality and variety of the subcellular localization labels in the training data, potentially leading to reduced performance in underrepresented or newly discovered subcellular compartments.

To further improve Loc4Lnc, future research can focus on incorporating additional biological contexts, such as RNA secondary structure and protein‐binding information, to provide a more comprehensive understanding of lncRNA functions. Moreover, exploring lightweight architectures or hybrid models could help reduce computational costs and make the model more accessible for broader applications. Finally, extending the model to support multilabel classification for lncRNAs localized to multiple subcellular compartments would increase its utility in understanding complex RNA localization patterns and their roles in diverse cellular processes.

## 4 MATERIALS AND METHODS

### 4.1 Overview of the RNALocate database

RNALocate v1.0 is an RNA subcellular localization database that has been widely used over the past decade, with a wealth of resources documenting more than 42,000 manually edited and experimentally evidenced RNA‐associated subcellular localization entries for more than 23,100 RNAs from 65 species and 42 subcellular localizations [34]. RNALocate provides a convenient user‐friendly interface for querying, browsing, and visualizing the details of the subcellular localization of these RNAs. Cui et al. released RNALocate v2.0 in 2021, which expands the number of RNA subcellular localization entries and integrates new datasets over the previous version [35].

**FIGURE 2:**
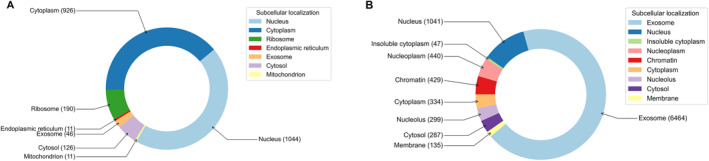
Comparative subcellular localization distributions between two versions of RNALocate database. (A) Distribution in RNALocate v1.0. (B) Distribution in RNALocate v2.0.

In the past few years, the benchmark datasets used by most researchers in conducting lncRNA subcellular localization studies were extracted from the database v1.0. However, we retrieved the distribution of lncRNA subcellular locations in the two databases and compared them, as shown in Figure 2 (few locations have been omitted). It can be seen that lncRNAs located in chromatin have not been documented in v1.0 but are recorded in v2.0. The first version of the RNALocate database showed quite a few lncRNAs in ribosomes but v2.0 deleted some of the records in ribosomes.

To improve and standardize subcellular localization prediction studies, we aim to create a benchmark dataset using the latest database version. This dataset is then used for training and testing models.

### 4.2 Dataset construction

The construction of the dataset consists of the following three steps.

1. We first concatenate all RNA subcellular location data corresponding to the biological species *Homo sapiens* in the RNALocate v2.0 database with the RNA sequences of humans provided in the database. Then, we filter the lncRNA subcellular location data of *Homo sapiens*. We extract 11,593 lncRNA subcellular location records from the database but some lncRNAs have the same Gene_ID in the database. After screening out the redundant entries based on the uniqueness of Gene_ID, we obtain 3444 data points. During this process, we also discard multilabel entries. These multilabel instances, which represented lncRNAs assigned to more than one subcellular location, were rare (less than 1% of the total dataset). Given the small proportion of multilabeled data and the potential complexity it would introduce to the model, we decided to exclude these instances to maintain dataset consistency and avoid unnecessary complications in the model training.
2. To address the problem of removing possible redundancy between sequences, we utilize CD‐HIT. The CD‐HIT program uses a novel parallelization strategy for the efficient clustering of biological sequence datasets, in which the CD‐HIT‐EST tool targets clustering nucleotides [36]. We use a cutoff rate of 80% and obtained 3322 entries.
3. This dataset of 3322 samples covers 13 subcellular location categories. As some of the subcellular locations have inclusion relationships with each other, we group some categories based on biological definitions. Specifically, we group nucleoplasm, nucleolus, and nuclear as nucleus locations [37]. Similarly, insoluble cytoplasm and soluble cytoplasm are merged as cytoplasm. By excluding the minor classes (less than 100 samples), we obtain five subcellular locations, namely exosome, chromatin, cytoplasm, cytosol, and nucleus.

**FIGURE 3:**
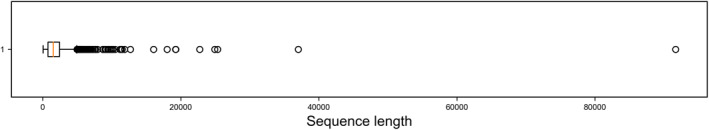
Distribution of the sequence length.

In summary, a total of 3277 lncRNA samples from five different subcellular locations constitute our benchmark dataset. The label distribution is shown in Table 4. Notably, these sequences exhibit a wide range of lengths, spanning from a minimum of 61 bp to a maximum of 91,671 bp. This considerable variation is reflected in the substantial standard deviation of 2368 bp. Figure 3 shows the distribution of the lengths of these sequences, which suggests the presence of some extraordinarily long sequences. Thus, we impose a sequence length threshold of 10,000 bp to remove exceptionally long sequences.

**TABLE 4:** Distribution of subcellular locations.

Subcellular location	Count
Exosome	2319
Chromatin	348
Cytoplasm	116
Cytosol	105
Nucleus	389
Total	3277

### 4.3 Feature extraction

**FIGURE 4:**
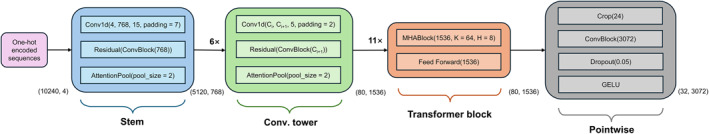
Architecture of the feature extraction process.

Long‐range interactions play a crucial role in understanding complex regulatory networks in DNA/RNA sequence prediction. However, traditional models struggle to efficiently capture these interactions due to computational complexity. Although transformer‐based models effectively handle long sequences, their complexity grows exponentially with sequence length. To balance the need for long‐range interaction capture and manageable computational complexity, we build a feature extraction model inspired by Enformer [30]. The overall model architecture we use to extract features of the lncRNA sequences is illustrated in Figure 4. It consists of four main components: the stem convolutional layer, the convolutional tower with 6 convolutional layers, 11 transformer blocks, and the pointwise layer.

#### 4.3.1 Input representation

The initial sequence is first padded to a length of 10,240 using *N* bases to represent unknown bases. The input to the model consists of one‐hot encoded RNA sequences, represented as a matrix $X_{0}\in R^{L\times D}$, where *L* denotes the sequence length (10,240 here, because the original sequences are all padded to 10,240 bp) and *D* represents the number of channels (4 in this case, each nucleotide is encoded as a four‐dimensional one‐hot vector, for unknown bases (*N*), we use a vector [0, 0, 0, 0] to represent uncertainty) [38].

#### 4.3.2 Stem module

The stem module consists of an initial convolutional layer followed by a residual block and an attention pooling layer. It primarily serves to perform low‐level feature extraction on the raw input, such as initial pattern recognition and local information capture, providing a solid foundation for deeper convolutional layers. The stem layer also plays a crucial role in transforming input dimensions by using carefully designed hyperparameters, such as a larger convolutional kernel size (15) and stride, to significantly reduce the sequence length while increasing the number of channels (from 4 to 768), thereby compressing the input features effectively. The use of a larger kernel size allows the stem module to cover a wider context window, ensuring that important initial features are captured without losing valuable information because of frequent pooling or convolution operations in the subsequent layers as the model depth increases.

The convolutional layer applies a 1D convolution operation to the input sequence *X*_0_, which is formally expressed as follows:

(8) $$\begin{array}{c} X_{1}^{\left(0\right)}=\sigma \left(X_{0}^{\left(0\right)}*W_{1}^{\left(0\right)}+b_{1}^{\left(0\right)}\right), \end{array}$$

where $W_{1}^{\left(0\right)}\in R^{k_{1}^{\left(0\right)}\times D\times C^{\left(0\right)}}$ is the convolution filter, $b_{1}^{\left(0\right)}\in R^{C_{0}}$ is the bias term, $k_{1}^{\left(0\right)}$ = 15 is the kernel size, and *C* ^(0)^ = 768 is the output channel dimension of the convolutional layer. ∗ represents the convolution operation. The activation function *σ* (ReLU in this case) is applied element‐wise.

Related deep learning studies have shown that directly adding the representation of one layer to the representation of the subsequent layer can help alleviate the vanishing gradient problem and improve the efficiency of gradient descent training [39]. Accordingly, a residual convolutional block is employed, where the input $X_{1}^{\left(0\right)}$​ is transformed as follows:

(9) $$\begin{array}{c} X_{2}^{\left(0\right)}=\sigma \left(X_{1}^{\left(0\right)}*W_{2}^{\left(0\right)}+b_{2}^{\left(0\right)}\right)+X_{1}^{\left(0\right)}, \end{array}$$

where $W_{2}^{\left(0\right)}\in R^{k_{2}^{\left(0\right)}\times C^{\left(0\right)}\times C^{\left(0\right)}}\;\text{is}$ the weight matric of the convolutional layer within the residual block with the kernel size $k_{2}^{\left(0\right)}=1$, and $b_{2}^{\left(0\right)}$ is the bias term.

Next, an attention pooling layer is applied with a window size of *L*_*p*_ = 2 and a stride of 2 to condense the sequence, effectively merging adjacent positions, shortening the sequence length, and reducing the computational load of the subsequent transformer layers:

(10) $$\begin{array}{c} X_{3,j}^{\left(0\right)}=\frac{\sum_{i}\exp\left(X_{2,i}^{\left(0\right)}\cdot w_{j}^{\left(0\right)}\right)X_{2,ij}^{\left(0\right)}}{\sum_{i}\exp\left(X_{2,i}^{\left(0\right)}\cdot w_{j}^{\left(0\right)}\right)}, \end{array}$$

where $X_{2,i}^{\left(0\right)}$ represents the feature vector at the *i*th position in the sequence of the feature matrix $X_{2}^{\left(0\right)}$, $X_{3,j}^{\left(0\right)}$ represents the vector at the *j*th position in the sequence of the output matrix $X_{3}^{\left(0\right)}$, $w_{j}^{\left(0\right)}\in R^{c^{\left(0\right)}}$ is a learnable weight vector, and $X_{2,ij}^{\left(0\right)}$ refers to the specific feature value at the *i*th position and *j*th channel which is used for the weighted sum. This operation reduces the sequence length by half, resulting in an output $X_{3}^{\left(0\right)}\in R^{L/2\times C^{\left(0\right)}}.$ $X_{3}^{\left(0\right)}$ is passed as the input $X_{0}^{\left(1\right)}$ to the next module.

#### 4.3.3 Convolutional tower

The convolutional tower consists of six identical blocks, each containing a convolutional layer followed by a residual block and an attention pooling layer. Each block applies a 1D convolution operation to the input $X_{0}^{\left(k\right)}$, where the output $X_{1}^{\left(k\right)}$ is computed as follows:

(11) $$\begin{array}{c} X_{1}^{\left(k\right)}=\sigma \left(X_{0}^{\left(k\right)}*W_{1}^{\left(k\right)}+b_{1}^{\left(k\right)}\right), \end{array}$$

where $W_{k}\in R^{5\times C^{\left(k-1\right)}\times C^{\left(k\right)}}$ is the convolution kernel for the *k*th block, and $b_{k}\in R^{C^{\left(k\right)}}$ is the corresponding bias. Similar to the stem module, the residual connection within each block is described as follows:

(12) $$\begin{array}{c} X_{2}^{\left(k\right)}=\sigma \left(X_{1}^{\left(k\right)}*W_{2}^{\left(k\right)}+b_{2}^{\left(k\right)}\right)+X_{1}^{\left(k\right)}, \end{array}$$

where $W_{2}^{\left(k\right)}\in R^{1\times C^{\left(k\right)}\times C^{\left(k\right)}}$ is the weight matrix of the convolutional layer within the residual block, $b_{2}^{\left(k\right)}$ is the bias term. Similar to the stem module, attention pooling is applied to the output of the residual block, enabling the model to capture long‐range dependencies across multiple convolutional layers.

#### 4.3.4 Transformer block

The transformer block comprises 11 layers of multihead attention (MHA) and feed‐forward neural networks designed to model long‐range dependencies between sequence positions. The input sequence is first projected into query, key, and value representations:

(13) $$\begin{array}{c} Q=XW_{q},K=XW_{k},V=XW_{v}, \end{array}$$

where *W*_*q*_,*W*_*k*_,*W*_*v*_ are the projection matrices [40]. The attention weights are computed as follows:

(14) $$\begin{array}{c} A=\text{softmax}\left(\frac{QK^{T}}{\sqrt{d_{k}}}+R\right), \end{array}$$

where $\sqrt{d_{k}}$ is a scaling factor to avoid large dot product values, *d*_*k*_ represents the dimensionality of the key vectors, and *R* = {*r*_*ij*_} represents the matrix of relative positional encodings with each element *r*_*ij*_ encoding the relative position between positions *i* and *j*. The attention output is calculated by applying the attention scores to the value matrix:

(15) $$\begin{array}{c} O=\mathrm{AV}. \end{array}$$

After MHA, a feed‐forward neural network is applied consisting of two linear layers:

(16) $$\begin{array}{c} \text{FFN}\left(X\right)=\max\;\left(0,XW_{1}+b_{1}\right)W_{2}+b_{2}. \end{array}$$

This block allows the model to capture complex nonlinear interactions in the sequence data.

#### 4.3.5 Pointwise module

After passing through the previous modules, the original sequence length of 10,240 bp is reduced to 80 bp by applying a pooling layer with a window size of 2, repeated 7 times (because each convolutional layer is followed by an attention pooling layer), effectively integrating every 128 bp into a single bin [41]. Following this, the pointwise module performs several sequential operations, including cropping, convolution, dropout, and the Gaussian error linear unit (GELU) activation function. The pointwise module is used to further refine and adjust the sequence by ensuring that the sequence length and feature dimensions are correctly aligned for downstream tasks. Specifically, in the pretrained model configuration, the target length is set to 32, and the cropping layer removes 24 bp from each side to achieve this target. The cropped output is then passed through a convolutional block to enhance feature representations, followed by a dropout layer with a rate of 0.05 to prevent overfitting. Finally, the GELU activation function introduces nonlinearity, allowing the model to capture more complex dependencies in the data as follows:

(17) $$\begin{array}{c} X_{\text{final}}=\frac{1}{2}X_{\text{drop}}\left(1+\tan\;h\left(\sqrt{\frac{2}{\pi }}\left(X_{\text{drop}}+0.044715X_{\text{drop}}^{3}\right)\right)\right), \end{array}$$

where the final output $X_{\text{final}}\in R^{L^{\prime }\times C_{\text{final}}}$ is the refined sequence embedding, *L*’ is the adjusted sequence length after cropping, and *C*_final_ is the number of output channels. We finally get the embedding vectors of size (32, 3072) for lncRNAs.

### 4.4 Classification

For the 5‐class classification task, we use TextCNN, which is a popular network for text classification tasks [31, 42]. The choice of three convolutional layers with filter sizes of 3, 4, and 5 allows the model to capture features of varying n‐gram lengths, and the *filter_number* is 100. The optimizer used is the Adam optimizer and the loss function uses the cross‐entropy loss function. The l*earning_rate* is set to 0.001 and the *batch_size* is 128. To avoid overfitting, a weight decay of 1*e*‐5 is used as part of the Adam optimizer settings, and a dropout rate of 0.5 is applied before the final fully connected layer.

## AUTHOR CONTRIBUTIONS

**Yujia Cheng**: Data curation; formal analysis; conceptualization; investigation; methodology; validation; visualization; writing ‐ original draft. **Xiaoyong Pan**: investigation; methodology. **Yang Yang**: Conceptualization; investigation; methodology; project administration; supervision; writing ‐ review and editing.

## CONFLICT OF INTEREST STATEMENT

The authors declare no conflicts of interest.

## ETHICS STATEMENT

This article does not contain any studies with human or animal subjects performed by any of the authors.

## Data Availability

All instructions, code, and specific experimental details, such as data preprocessing steps and parameter settings, can be found on our GitHub repository at tourbiIIon/Loc4Lnc.
